# Investigating the Impact of Augmented Reality Instruction Modes for Manual Wire Harness Assembly Task on Formboards

**DOI:** 10.3390/bs16071066

**Published:** 2026-06-29

**Authors:** Junfeng Wang, Jiang Zhan, Qifeng Zou, Yufan Lin, Lei Wu

**Affiliations:** 1 Department of Industrial and Manufacturing Systems Engineering, School of Mechanical Science and Engineering, Huazhong University of Science and Technology, Wuhan 430074, China; wangjf@hust.edu.cn (J.W.); m202470603@hust.edu.cn (J.Z.); 2 Department of Industrial Design, School of Design, Huazhong University of Science and Technology, Wuhan 430074, China; m202470464@hust.edu.cn (Q.Z.); m202270435@alumni.hust.edu.cn (Y.L.)

**Keywords:** augmented reality, assembly instruction, wire harness, gaze behavior, perceived workload, task performance

## Abstract

Wire harness assembly is a highly manual job performed on formboards. Augmented reality (AR)-assisted wiring operations can improve work efficiency and reduce operator workload. However, investigations into the effects of AR-assisted wiring assembly on operator performance remain in the preliminary stage. To investigate how different AR wire harness modes support novice operators in completing assembly tasks effectively, this exploratory laboratory study examined the impacts of AR instruction modes for single-route conditions on assembly performance (task time and number of assembly errors), gaze behavior using eye-tracking data, and subjective experience measured with the NASA-TLX (Task Load Index) as a post-experiment questionnaire in a controlled laboratory environment. Three wire path visualization modes, i.e., static color mode (SCM), dynamic color mode with flashing display (DCM-FD), and dynamic color mode with segment display (DCM-SD), were implemented for monitor-based, AR-assisted wiring instruction on a formboard. The results reveal a substantial influence of the wire path visualization modes on task time under our controlled experimental conditions: the SCM group achieved an 18% shorter task time than the other two groups, with a statistically significant difference. This finding contradicts the existing observations in the mechanical assembly domain. For gaze behavior, an analysis of the eye-tracking data indicated that the number of switches in the SCM group was the lowest among the three groups, with a marginally significant difference from the DCM-FD group for both low- and high-complexity wiring tasks during the laying phase. Additionally, the total fixation time of the three groups showed a significant difference for low- and high-complexity tasks with a large effect size; the SCM group exhibited the shortest total fixation time across all tasks. No significant differences in the number of assembly errors and users’ perceived workload were observed among the three groups. These findings can serve as a reference for guiding the visual style design in AR-assisted wiring systems for training novice operators in human-centric Industry 5.0 and achieving a decrease in overall workload and improved task performance.

## 1. Introduction

**Figure 1:**
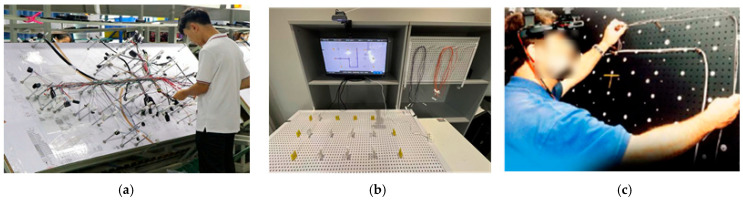
Wire-harness assembly operation on formboard: (**a**) traditional paper-based operation; (**b**) monitor-based augmented reality operation in our experiment; and (**c**) head-mounted display (HMD)-based operation from Caudell and Mizell (1992).

Traditionally, wire harnesses are assembled on a custom-made formboard or nailboard (Caudell & Mizell, 1992; Rice et al., 2016; J. Wang et al., 2023). The operation instructions are paper-based schematics attached to the formboard (Figure 1a), which is a type of ‘template’ for the assembly of wire bundles. There are also many wire list manuals showing textual wire data, such as wire numbers, types, and clamp locations and laying paths. The operator usually spends considerable time searching for the required information and switching their attention between paper-based manuals and the assembly formboard. Wiring operations are operator-intensive, time-consuming, and error-prone work due to the large number of wires and the increasing customizability of wire harnesses for the final mechatronic products (Luque et al., 2022; Szajna et al., 2020). The diversity, complexity, and flexibility of various wires pose substantial challenges to automatic installation, meaning wire harness assembly remains highly dependent on manual operation (Nguyen et al., 2021).

Augmented reality (AR) facilitates information overlaying within the users’ field of view to assist in their tasks (Figure 1b,c). AR-assisted assembly (ARAA) offers sequential visual instructions, emphasizing how to recognize tools/parts/materials, where to obtain them, and how to apply them in assembly operations (Gattullo et al., 2022; Z. Wang et al., 2022). The AR-based information support system is effective in decreasing assembly errors and improving work efficiency, when compared with other types of instructional methods (Cammeraat et al., 2020; Giridhar et al., 2020; Lavric et al., 2022; Stockinger et al., 2021; Rice et al., 2016; Howard et al., 2023; X. Wang et al., 2016). While several studies also have reported that AR-assisted work is frequently associated with time expenditure, discomfort, and increased workload (Botto et al., 2020; Min et al., 2020; Kaplan et al., 2021; Drouot et al., 2022), the efficacy of AR as an assembly assistance tool remains contentious.

Currently, few ARAA studies focused on the wire harness assembly (Daling & Schlittmeier, 2024). The impacts of the wire path visual style on operator information searching behavior and task performance are under-investigated. We therefore proposed the following two research questions (RQs):

RQ1: How do various AR wire harness visualization modes differ in directing visual attention and supporting task performance during wiring operations?

RQ2: How do various AR wire harness visualization modes individually affect users’ subjective experience in wiring assembly tasks?

To address these questions, this study conducted controlled laboratory experiments to train novice operators in wire harness assembly. Three instruction types, i.e., static color mode (SCM), dynamic color mode with segment display (DCM-SD), and dynamic color mode with flashing display (DCM-FD), were evaluated based on an objective measure (eye-tracking data), performance (task time and assembly errors), and a subjective measure (NASA-TLX). In SCM, a static, color-coded path was overlaid on the real-time video of the formboard, matching the wire’s color to indicate the laying path. The DCM-SD was a segment-dynamic sequential display, where the wire path model was displayed segment by segment according to a predefined animation cycle. The dynamic global DCM-FD displayed the wire path model in full and repeatedly flashed bold to double the wire width. This study’s findings can provide baseline evidence of the ARAA’s impact on assembly training scenarios for novice wire harness operators, rather than offering direct recommendations for workstations operated by skilled personnel. The results can also serve as a reference for AR interface designers and programmers.

## 2. Related Work

Although many research studies have shown that the ARAA method can successfully reduce task time and error rates compared with paper-based manual methods, full AR adoption in assembly activities remains a challenge (Kim et al., 2018; Jetter et al., 2018; Dreesbach et al., 2023; Fang et al., 2025; Navas-Reascos et al., 2026). A primary barrier is usability, as current ARAA implementations do not yet optimize the visualization design of instructional content for different virtual assets (Stork & Schubö, 2010; Abubakar & Wang, 2019; Schuster et al., 2021). Deploying ARAA system designs based on trial and error without experimental verification can degrade task performance, increase workload and distraction, and even expose operators to occupational hazards, thus undermining user trust (Endsley et al., 2017; Masood & Egger, 2019; Kaplan et al., 2021; Buchner et al., 2022). How to present AR information for assembly users has thus become an important topic in academic and industrial research in recent years.

### 2.1. Augmented Reality in Wire Harness Assembly Activities

A wire harness usually contains hundreds or even thousands of wires. Modern wire harness companies typically use a transferred assembly line to produce the wire assembly. In a workstation, manual wiring is conducted according to comprehensive printed paper documents. These documents illustrate electrical diagrams and methods of wire connections. A professional electrician carefully analyzes every page of the assembly drawings and fits individual wires one by one on the wiring board. The wire harness industry now strives to achieve increased manual work efficiency via digital assembly instruction methods (J. Wang et al., 2023).

As early as 1992, the concept of “augmented reality” was first proposed by Boeing for wiring harness assembly on formboards, with the company reporting a 25% reduction in assembly cycle time and a near-zero error rate (Caudell & Mizell, 1992). Rice et al. (2016) developed a wire harness guidance system that integrated mobile and wearable devices. By comparing three visual interface designs (i.e., Text, AR, and a 2D Schematic view), the user study showed the higher performance of graphical representation over text-only information in wire assembly guidance on the formboard. Szajna et al. (2020) described an ARAIS for wire assembly and the control cabinet production process using HoloLens. The virtual elements (arrow, dashboard, etc.) were set and adjusted for the augmented visualization of the wring process. They tested the system by performing a 20-wire assembly task and the complete assembly time decreased by 31% compared with non-AR operation. Papetti et al. (2022, 2023) evaluated four user experiences for wire assembly and quality control including the paper-based manual, monitor-based display, mixed reality with an HMD, and projection-based AR. The experiments showed that the projection-based AR method achieved a shorter assembly completion time (10.9 ± 3.8 min) and the monitor-based display method yielded the fewest assembly task errors (1.6 ± 1.9).

To compare the effects of AR visual elements, Zhao et al. (2021) devised a case featuring four assembly steps for cable assembly within a cabinet. The NASA-TLX outcomes indicated that AR was most effective in decreasing the operator’s mental workload and increasing the assembly performance. The participants assigned the highest satisfaction to symbol instructions and the lowest to model instructions. In a trunking routing task scenario, Fang et al. (2024) and T. Zhang et al. (2025) experimented with a tablet-based AR system to perform a simple wiring task (three wires) and complex task (twenty wires). The AR instruction system differed significantly compared with a paper wire sheet regarding assembly completion time and TLX scores. The use of HoloLens for large-scale wire-laying tasks (Geng et al., 2024) also revealed that the wire-laying efficiency improved by 27.21% when comparing the AR method with a traditional wire list manual. In a study on operator training for wire harness activities over a tractor’s drivetrain (Brunzini et al., 2024), user errors decreased when comparing explanatory videos in anchor-based AR featuring animated CAD model AR instruction. For the user experience, 90% of operators reported that AR instruction promoted concentration while performing tasks.

It can be concluded that the current research predominantly applies objective methods to evaluate the task load during AR-assisted wire harness assembly. There are also few studies examining the instruction effectiveness of different virtual assets during the wire harness ARAA.

### 2.2. Effectiveness and Cognitive Issues of Virtual Assets in AR-Assisted Assembly

ARAA enables various visual assets to be displayed in the mixed view of operators, ensuring a clearer communication of the assembly process (W. Li et al., 2019). Considering the virtual assets in ARAA, there are many different kinds of virtual assets used in the literature, such as the text, picture, static, or dynamic model, and video (Gattullo et al., 2022). In human factor fields under the Industrial 4.0/5.0 perspective (Buchner et al., 2022; Fang et al., 2025), concerns over cognitive issues using virtual assets focused on identifying which types of virtual assets are more effective in transmitting specific assembly information, such as the position, orientation, direction, operation, and process, to operators under varying assembly task conditions (Abubakar & Wang, 2019; Giridhar et al., 2020; Forero et al., 2022; B. Wang et al., 2024). Currently, although some guidelines have been proposed for virtual asset design in ARAA (Agati et al., 2020; Jeffri & Rambli, 2020), no consensus has been reached in the literature on the most effective method of information presentation and instruction provision for users (Tainaka et al., 2023).

There are few studies on AR-assisted wire harness assembly, with most studies focusing on the structural components of products (Hoover et al., 2020). The ergonomic aspects of AR-assisted assembly instruction are influenced by technical factors (X. Wang et al., 2016; Z. Wang et al., 2022; Eswaran et al., 2023; J. Wang et al., 2024), human factors (Kaplan et al., 2021; Buchner et al., 2022; Chaudhari et al., 2025), assembly environment/objects (Suzuki et al., 2024; V. Zhang et al., 2024), and ways of displaying instructional content (Szajna et al., 2020; Danielsson et al., 2021; Atici-Ulusu et al., 2021).

Virtual assets/elements can represent the what, where, how, and why information for AR-assisted assembly instruction (W. Li et al., 2019; Gattullo et al., 2022) through text, videos, pictures/drawings, signs/arrows, 3D static models, and 3D animation. Designers must ensure that element selection aligns with the assembly task at hand (Alenljung & Lindblom, 2021). It is therefore important to understand how different visualization types influence user work considering the workload and performance.

Compared with two-dimensional drawings, Hou et al. (2013, 2015) demonstrated that dynamic AR visualization yielded a shorter task completion time (50% reduction), fewer assembly errors (50% reduction), and a lower total task load. In a Lego bricks assembly scenario (Smith et al., 2020), an in situ video-based AR instructions method allowed users to complete the task an average of 21% faster than those using CAD models superimposed onto the environment; it also led to significantly fewer errors than using annotated models. Using 3D animation mode led to a significantly faster completion time compared with video-based visualization (Ariansyah et al., 2022). For the part orientation task, Laviola et al. (2025) found that the instructing method of animated side-by-side product models resulted in the shortest completion time, leading to less cognitive load considering the fixation time, number of fixations, and subjective mental effort. The HMD-based ARAA method required operators to spend more time reading and processing textual information compared with traditional training materials (X. Yang et al., 2023). In ARAA, only the minimum amount of information required by the assembly task should be presented. Laviola et al. (2024, 2025) proposed minimal AR to optimize the displayed information and reported that the redundant AR information could not improve task comprehension. The simplified 3D models also did not impact the user experience during the ARAA process (Arige et al., 2022).

The combined display of multi-type virtual assets can give full play to the characteristics of different assembly information categories. Moghaddam et al. (2021) showed that some participants found the combination of text and 3D CAD animation more helpful than the expert capture videos during a fuel cell module assembly process. Concrete instructing information (e.g., 3D product models and video) did not provide a significant advantage to abstract instructing information (e.g., wire mesh boxes model, 3D arrows, shapes, lines, and frames) regarding completion time, but they lowered the overall assembly error rate (Radkowski et al., 2015; Jasche et al., 2021). For repetitive mechanical rigid-part assembly tasks, users exhibited a preference for more detailed and intuitive HMD-based AR instructions, e.g., text, arrows, highlighting information, and the overlaying model on the physical object (J. Li et al., 2024).

### 2.3. Research Hypotheses

In the wire harness flow assembly mode, each participant of a formboard workstation is responsible for placing a certain number of wires. The workstation time is limited by the production cycle, which requires operators to lay the wires in place as quickly as possible.

For wiring operations, numerous studies showed that ARAA can improve task performance compared with paper-based manual methods (Rice et al., 2016; Szajna et al., 2020; Papetti et al., 2022, 2023; Marino et al., 2024; Fang et al., 2024; Geng et al., 2024; Brunzini et al., 2024; T. Zhang et al., 2025). However, the existing literature has not yet investigated the differences in effectiveness across various AR modes for wiring processes. Rigid-part mechanical assembly processes focused on 3D spatial perception for the positioning and assembly of standard rigid parts. Several studies have shown that the visual styles of AR modes had diverse impacts on the user’s performance and workload (Ariansyah et al., 2022; Laviola et al., 2025; J. Li et al., 2024; Moghaddam et al., 2021; Gattullo et al., 2022). Meanwhile, the wiring operation centered on complex routing paths on 2D planar formboards. These differences prevent the research findings on AR visual effects in mechanical assembly from being applied to wiring operations.

This study therefore conducted a comparative evaluation of wire harness assembly processes, investigating the presence of significant differences in the task performance, gaze behavior, and perceived workload under different visual styles of AR modes. Training novice operators in wire harness assembly is essential for improving the task performance and reducing the workload. Considering the two proposed RQs, this study proposed the following four research hypotheses:

**H1.**  *The visual style of the wire harness mode has a significant effect on the task time during the ARAA process for novice operators.*

**H2.**  *The visual style of the wire harness mode has a significant effect on the number of assembly errors during the ARAA process for novice operators.*

**H3.**  *The visual style of the wire harness mode has a significant effect on the gaze behavior during the ARAA process for novice operators.*

**H4.**  *The visual style of the wire harness mode has a significant effect on users’ subjective experience for novice operators.*

## 3. Methods

### 3.1. Assembly Task and Apparatus

To closely replicate on-site operation, an eight-wire harness assembly was designed considering the takt time of a formboard workstation. The eight wires consisted of two color types (Table 1), and each wire had different number of segments. A segment was defined as a single straight section of wire with no bends or folds after routing. Clamps were used to fasten the wires along the routing path. The wiring configurations of all eight wires are shown in Appendix A (Table A1). Currently, there is no unified and widely accepted standard definition of task complexity for mechanical or wire harness assembly tasks in AR research (J. Wang et al., 2024). For mechanical products, the complexity of assembly tasks is related to the number of parts with degrees of freedom for part orientation (Bläsing & Bornewasser, 2021), part shape similarity (J. Li et al., 2024), the number of similar parts (Z. Yang et al., 2019), and part size/weight (Chiew & Sung, 2022). For wire harness assembly, only the number of wires was used to classify the task complexity in the AR study (Fang et al., 2024), where the low-complexity task consisted of completing the assembly of three wires and the high-complexity task involved assembling twenty wires. For the repetitive complex assembly task, more attention is paid to the impact of individual step operations on the overall process (J. Li et al., 2024). We therefore defined the assembly task for wires 1, 3, 4, and 6 as complex tasks, as they involved relatively more clamps (seven clamps) on the wire path. Each wire had a different laying path which was constrained by several clamps. All participants performed the wire harness assembly task in the same fixed order based on the wire number (see Table 1).

**Figure 2:**
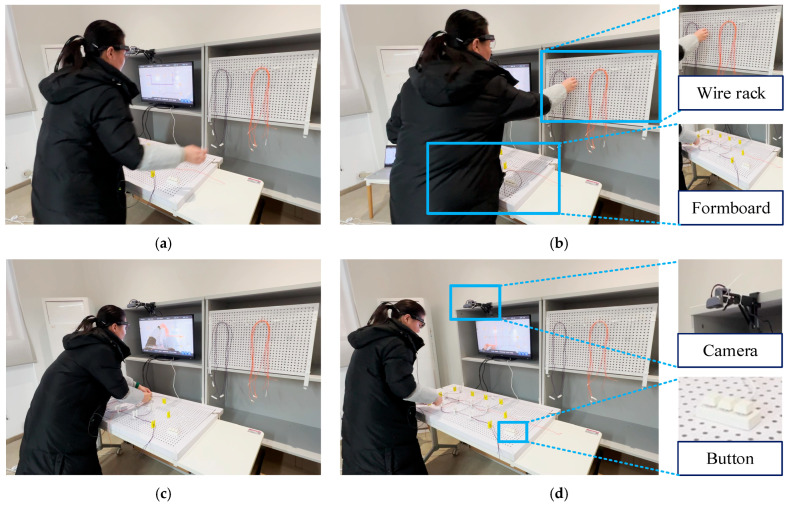
The two sub-tasks of the wire assembly process: (**a**) looking at screen in matching task; (**b**) taking a wire in matching task; (**c**) looking at screen in laying task; and (**d**) deploying a wire in laying task.

The layout of the experimental workstation and the assembly process tasks are shown in Figure 2. The workstation had a white-colored formboard (60 × 120 cm size), a wire rack, a camera, a monitor, and a three-key button. Before the experiments, some clamps were installed on the formboard to restrict the wire path. AR instructions for wires and their paths were displayed on a 27-inch LCD monitor with a resolution of 3840 × 2160 pixels and 60 FPS (Frames Per Second). The real-time video was captured using a 4-million-pixel camera with 1920 × 1080 resolution output at 30 FPS. Tobii Pro Glasses 2 was used to record the eye movement data at a sampling rate up to 100 Hz.

We chose a monitor-based AR instruction method due to its advantages of being hands-free and requiring no physical body load, which is more easily accepted by shop floor operators. To simplify AR system development for laying the 2D wire harness on the formboard, we built the AR scene generation scheme based on the ‘Cameo’ feature in Microsoft 365 PowerPoint (Microsoft, 2022). We initially established color-coded 2D wire paths on PPT slides with reference to the wiring formboard size and wire layout scheme. Real-time camera video streams were imported into PPT via the Cameo feature and set as the bottom layer, while the predefined 2D wire harness path models were set as the upper layer. Subsequently, a live AR view was rendered in PPT and shown on the display monitor. By adjusting the camera height and focal length, the wire path models were aligned with the physical wiring formboard.

The wire assembly process comprised two sub-tasks, i.e., matching and laying task (Figure 2). In the matching task, the participant first looked at the screen to ascertain the wire’s color and then picked up the wire from the rack. In the laying task, the participant looked at the screen to learn the path of the wire and laid the wire on the formboard according to its path. Looking at the screen for the required wire assembly information was treated as a cognitive process (Papetti et al., 2023; Fang et al., 2024). Selecting the right wire and laying it on the formboard were classified as task execution behaviors. For complex tasks, the participant may look at the screen several times while deploying the wire on the formboard. During the two phases, the experimenter recorded the assembly errors of the participants.

### 3.2. Experimental Design and Measures

**Figure 3:**
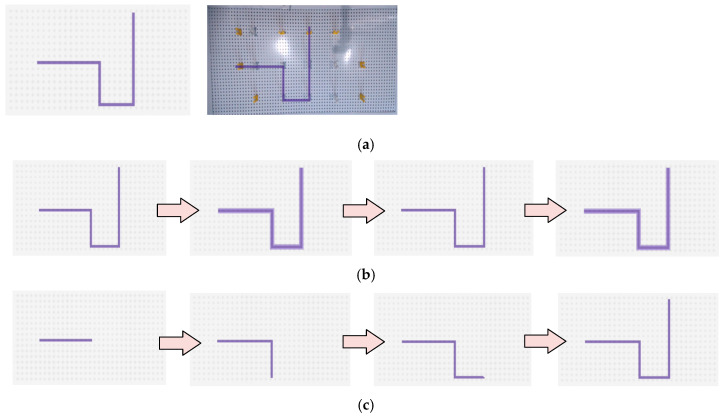
Three visual modes for a wire assembly instruction: (**a**) SCM; (**b**) DCM-FD; and (**c**) DCM-SD.

The experiments adopted a between-subject design, with three experimental conditions to which participants were randomly assigned. Previous studies on cognitive load and experimental research in industrial applications of ARAA guidance (Giridhar et al., 2020; Hou et al., 2013, 2015; Laviola et al., 2025; Jeffri & Rambli, 2020; Lušić et al., 2016; Suzuki et al., 2024; Z. Yang et al., 2019) have shown that static and animated modes serve as two key types of instruction in AR applications. We therefore designed three types of wire visualization modes, i.e., SCM, DCM-FD, and DCM-SD, for presenting assembly instructions. The two selected dynamic modes represent the two most typical dynamic AR guidance paradigms in current assembly research. Figure 3 shows the path of the first wire in Table 1 with three visual modes. After completing the laying of the current wire, the participants pressed a button and the system showed the path of the next wire.

After preliminary experiments, the opacity of the wire path model was set to 60%. The assembly formboard had holes with a 6 mm diameter and a hole pitch of 16 mm. The thickness of the AR wiring harness model was chosen to be 2 times the hole diameter, i.e., 12 mm, to ensure good visibility and the accurate position indication. For the animation parameters of the dynamic modes, the path model was set to flash for 2 s per cycle and the segment progression speed was configured at 25 cm/s referencing the existing literature (Laviola et al., 2025; Roberts, 2012; Wickens et al., 2021). Taking each hole’s center point and the corresponding points on the centerlines of 2D wire models as sample points, we calculated the RMSE from their pixel coordinates in the AR view. Under the present experimental setup, the AR overlay alignment achieved an RMSE (Root Mean Square Error) of 2.789 mm, which is acceptable for AR-assisted wire harness assembly considering the hole pitch (16 mm) and clamp opening size (10 mm) in our experimental setup. The frame rate of AR video was close to 30 FPS. The AR overlay remained stable in position and clarity across all conditions. These settings ensured that the observed differences in task performance and gaze behavior could be attributed to the wire-path display modes rather than artifacts introduced by the AR system.

During the experiments, the task time and the number of assembly errors were recorded, which reflect the overall task execution performance in the AR-assisted wire assembly process. For the assembly errors, we counted three types of errors in each experiment. A Type I assembly error referred to the participant selecting a wire whose color did not match the instructed color. A Type II assembly error was defined as the participant routing the wire through a non-required clamp. A Type III assembly error was defined as the participant omitting a required clamp during wire routing. Eye movement measures were used to explore the different strategies utilized by the users to acquire the information required to perform the task, which demonstrate visual attention allocation of operators. To capture eye-gaze behavior, the study converted the collected raw data into three common parameters: number of switches between Areas of Interest (AOIs), fixation time for an AOI, and number of fixations. The number of switches between AOIs was calculated as the total count of gaze transitions from one AOI to another. Three AOIs were considered (rack, display screen, and formboard) in our study. After completing each experimental trial, participants were required to fill out a questionnaire about their experience, in which the NASA-TLX was used to assess their subjective task load perception based on a standard questionnaire.

### 3.3. Participants and Procedure

Participant recruitment was conducted through both online and offline methods. Information about the study was spread via university-related social media platforms and promoted by distributing materials in public areas on the university campus or through word-of-mouth recommendations. The recruited participants needed to satisfy the following criteria: be mentally and physically healthy, not have any psychological or neurological disorders (e.g., anxiety, epilepsy, or depression), have normal vision or corrected-to-normal vision, and have no color-blindness or color-weakness.

A total of 45 participants were recruited for this experiment, all of whom were undergraduate or postgraduate students at our university, with 13 male (28.9%) and 32 female (71.1%) participants. Their ages ranged from 18 to 27 years (M = 22.64, SD = 2.11). Regarding the level of education, there were 16 undergraduate students (35.6%) and 29 graduate students (64.4%). All participants had no wire harness assembly experience and were treated as novice operators. Before the experiment, every participant was fully informed about the study and signed an informed consent form. After the experiment was completed, each participant was given a compensation of thirty yuan.

The experiment was conducted in our university laboratory. The overall procedure had four stages. First, the experimental apparatus, the wire harness assembly tasks, and the monitor-based AR instruction were introduced. The experimenter demonstrated the assembly process and the participants were allowed to ask questions related to the experiment. Second, the participants were helped with wearing the eye-tracking device. Debugging and the calibration process were performed for each participant. Participants engaged with the wire harness assembly exercises based on the AR instructions step by step. After completing an assembly step, they needed to press a button on the table to proceed to the next step until all wires were laid on the formboard. Third, the formal assembly experiment began. Participants were required to strictly adhere to the given instructions without any assistance from the test supervisor throughout the assembly process. The experimenter monitored the participants and recorded the number of assembly errors, which was the total number of Type I to III assembly errors for all participants in each group. Each error was recorded once as it occurred, regardless of whether it was subsequently detected and corrected by the participant. The error counts were verified and checked using post hoc video review. Last, participants were asked to rate their perceived workload via the NASA-TLX survey. The experiment lasted approximately 15 min for each participant. All participants performed eight assembly tasks in the identical sequence. To reduce potential practice effects, all participants received sufficient pre-experiment training to get familiar with the operations before formal testing, which largely eliminated the progressive learning effect during the formal trials. In addition, the eight wire-laying tasks differed obviously regarding start/end clamp and wire routing, so familiarity gained from early tasks could not be directly transferred to subsequent ones.

## 4. Results

In this study, the independence of observations was ensured by completely randomly assigning participants to three AR mode groups. During data analysis, the normality and homogeneity of variance were first verified via the Shapiro–Wilk (suitable for small sample sizes in this study) and the Levene tests, respectively. If the normality and homogeneity of variance were all confirmed, a one-way ANOVA was used to test the overall main effect among the three groups, and then the Tukey’s Honestly Significant Difference (HSD) method was used for post hoc multiple comparisons. If the normality assumption was not satisfied, the Kruskal–Wallis test was adopted and then pairwise comparisons were performed using the Dunn test with Bonferroni correction for multiple testing. If the normality assumption was satisfied but the homogeneity of variance assumption was not, a Welch ANOVA was adopted to test the overall main effect, and Tamhane’s method (for a small, imbalanced sample and unequal variances) was used for post hoc multiple comparisons.

This study set the predetermined statistical significance level at *p* < 0.05. Error bars in the graphs correspond to a 95% confidence interval, where * denotes *p* < 0.05, ** represents *p* < 0.005, and *** means *p* < 0.001. Tobii Pro Lab was used for eye-tracking data processing. After screening and removing eye movemental experiment data with low sampling rates (less than 70% in Tobii Pro Lab), valid eye gaze data were gathered for 11, 10, and 12 participants in the SCM, DCM-FD, and DCM-SD groups, respectively. The task performance, gaze behavior, and perceived workload were analyzed based on data from 33 participants considering the equilibrium of the sample sizes. The main descriptive statistics for dependent variables across the three AR conditions are shown in Table 2. To address the small, unbalanced sample sizes, a more conservative post hoc test method was used, and the effect sizes ($\eta_{p}^{2}$ and Cohen’s d) and their 95% confidence intervals (CIs) were reported.

### 4.1. Task Performance

#### 4.1.1. Task Time

**Figure 4:**
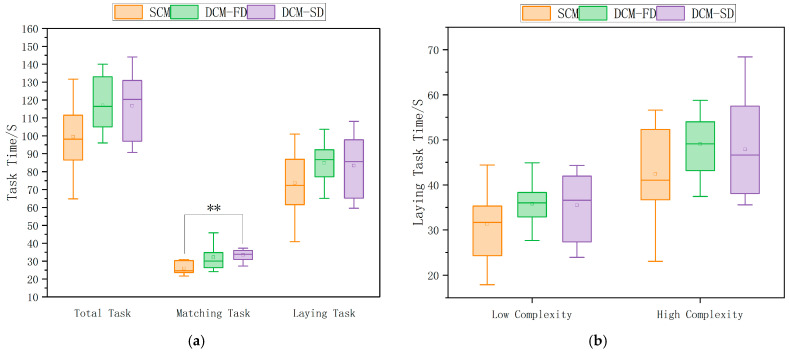
Assembly task time of harness wiring tasks in three groups: (**a**) assembly tasks of all wires; and (**b**) low- and high-complexity laying tasks. ** represents *p* < 0.005.

The total assembly task time in this study refers to the time from when participants start to when they finish the assembly tasks for all eight wires, including the wire matching task time and laying task time. Figure 4 shows the box plot of the average assembly time of tasks across the three groups.

A one-way ANOVA revealed a significant main effect of visualization modes on the total assembly task time—F(2, 30) = 3.431, *p* = 0.046, $\eta_{p}^{2}$ = 0.186—with a large effect size. However, Tukey’s HSD post hoc tests revealed no significant pairwise differences between any of the three groups (all *p* > 0.05). As shown in Table 2 and Figure 4a, the assembly task is completed fastest on average in the SCM group (mean = 99.555), compared with the DCM-FD group (mean = 116.987) and the DCM-SD group (mean = 116.812).

For the matching task involving the assembly of all wires, the matching time of the three groups did not pass the Levene test (F(2, 30) = 3.807, *p* = 0.034 < 0.05). A Welch ANOVA was used to examine the main effect, and the results reveal a significant main effect of AR wire modes on matching task time, F(2, 30) = 14.974, *p* < 0.001, $\eta_{p}^{2}$ = 0.357, 95% CI [0.075, 0.536]. A post hoc test based on Tamhane’s method shows that a significant difference is detected in the matching task time between SCM and DCM-SD (mean difference = −7.637, SD = 1.389, Cohen’s d = −1.614, 95% CI [−2.63, −0.60], *p* = 0.002). No significant difference in laying task time is observed among the three groups, F(2, 30) = 1.648, *p* = 0.209, $\eta_{p}^{2}$ = 0.099, 95% CI [0.000, 0.265].

Figure 4b and Table 3 show the statistics of assembly time with laying task complexity. A one-way ANOVA showed no significant differences among the three groups in both low-complexity tasks (F(2, 30) = 1.416, *p* = 0.259, $\eta_{p}^{2}$ = 0.086) and high-complexity tasks (F(2, 30) = 1.571, *p* = 0.225, $\eta_{p}^{2}$ = 0.095), with a moderate effect size.

#### 4.1.2. Number of Assembly Errors

A total of six assembly errors were detected during the wire assembly task across 33 participants: two errors in the SCM group, three errors in the DCM-FD group, and one error in the DCM-SD group. All the errors were found and solved by the participants themselves. As the data does not satisfy the assumptions of normality and homogeneity of variance, a Kruskal–Wallis test was conducted. The results show no significant differences among the three groups with a small effect size (H(2) = 1.669, *p* = 0.434, ε^2^ = 0.018, 95% CI [0.000, 0.068]).

### 4.2. Gaze Behavior

#### 4.2.1. Number of Switches Between AOIs

As shown in Table 2, a one-way ANOVA showed no statistically significant effect (F(2, 30) = 3.139, *p* = 0.058, $\eta_{p}^{2}$ = 0.173) of the wire visualization mode on the number of switches across three groups during the whole assembly process of eight wires, where three AOIs were considered (rack, display screen, and formboard).

Further analysis revealed a significant difference in the number of switches during the laying phase among the three groups based on one-way ANOVA (F(2, 30) = 3.561, *p* = 0.041, $\eta_{p}^{2}$ = 0.192, 95% CI [0.003, 0.380]). Post hoc comparisons with Tukey’s HSD test showed that the number of switches in the SCM group (mean = 57.727, SD = 17.315) is significantly lower than that for the DCM-FD group (mean = 74.900, SD = 11.298) during the laying tasks (mean difference = −17.173, SD = 6.450, Cohen’s d = −1.168, 95% CI [−2.11, −0.22], *p* = 0.031).

**Figure 5:**
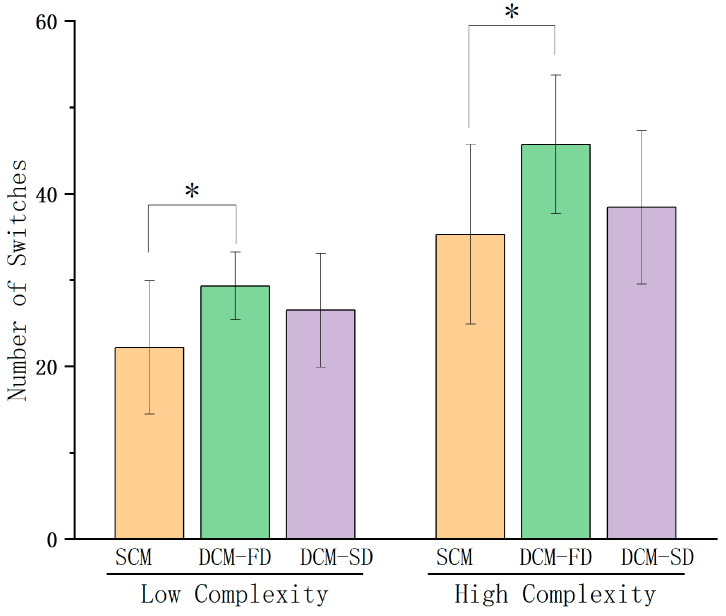
Number of switches in laying task with different task complexity. * denotes *p* < 0.05.

Considering the laying task complexity, the number of switches between two AOIs, i.e., display screen and formboard, of the three groups was significantly different (see Table 4 and Figure 5 for the one-way ANOVA results with effect sizes and CIs). Post hoc comparisons with Tukey’s HSD test showed that the number of switches for the SCM group (mean = 22.182, SD = 7.718) was significantly lower than that for the DCM-FD group (mean = 29.300, SD = 3.917) in the low-complexity laying tasks (mean difference = −7.1118, SD = 2.780, Cohen’s d = −1.119, 95% CI [−2.057, −0.179], *p* = 0.041). In the high-complexity laying tasks, the number of switches in the SCM group (mean = 35.272, SD = 10.403) also differed significantly from that for the DCM-FD group (mean = 45.700, SD = 8.028), with a mean difference = −10.427 (Cohen’s d = −1.136, 95% CI [−2.040, −0.232], *p* = 0.037). Cohen’s d indicates a large effect size, suggesting a potentially substantial difference between the two groups.

#### 4.2.2. Fixation Time on the AR Display

**Figure 6:**
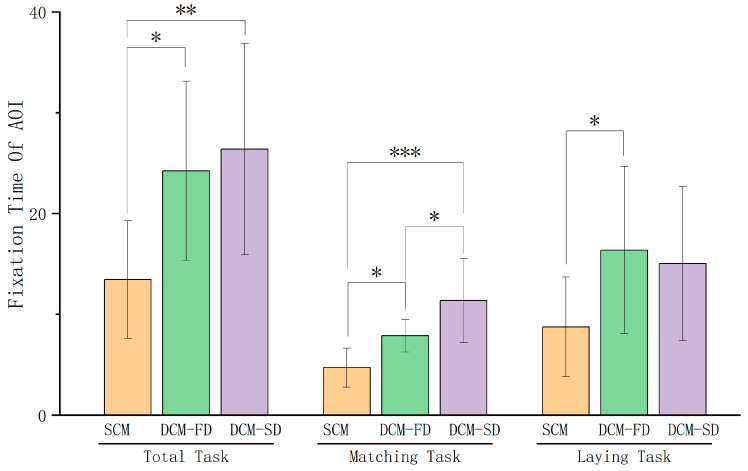
Fixation time of three groups for the total task and the two sub-tasks. * denotes *p* < 0.05, ** represents *p* < 0.005, and *** means *p* < 0.001.

As shown in Table 2, a statistically significant difference (*p* = 0.003) in total fixation time on the display screen was observed for the three groups according to the one-way ANOVA (F(2, 30) = 7.123, $\eta_{p}^{2}$ = 0.322). Post hoc analysis with the Tukey’s HSD test revealed that the total fixation time of the SCM group (mean = 13.436, SD = 5.842) was significantly lower than that of DCM-FD group (mean = 24.217, SD = 8.901, mean difference = −10.781, Cohen’s d = −1.241, 95% CI [−2.19, −0.29], *p* = 0.021) and DCM-SD group (mean = 26.386, SD = 10.488, mean difference = −12.950, Cohen’s d = −1.491, 95% CI [−2.429, 0.552], *p* = 0.003), as shown in Figure 6.

A one-way ANOVA analysis was conducted on the fixation time in the matching task (F(2, 30) = 15.096, *p* = 0.000, $\eta_{p}^{2}$ = 0.502, 95% CI [0.206, 0.648]) and laying task (F(2, 30) = 3.566, *p* = 0.041, $\eta_{p}^{2}$ = 0.192, 90% CI [0.005, 0.352]), where a significant effect was observed with a large effect size. According to Tukey’s HSD test, the fixation time during the matching task in the SCM group (mean = 4.699, SD = 1.934) was significantly lower than that in the DCM-FD (Cohen’s d = −1.087, 95% CI [−2.024, −0.149], *p* = 0.048) and DCM-SD (Cohen’s d = −2.291, 95% CI [−3.335, −1.246], *p* = 0.000) groups, while the fixation time during the laying task in the SCM group (mean = 8.737, SD = 4.938) was significantly lower than that in the DCM-FD group (Cohen’s d = −1.075, 95% CI [−2.012, −0.139], *p* = 0.049).

**Figure 7:**
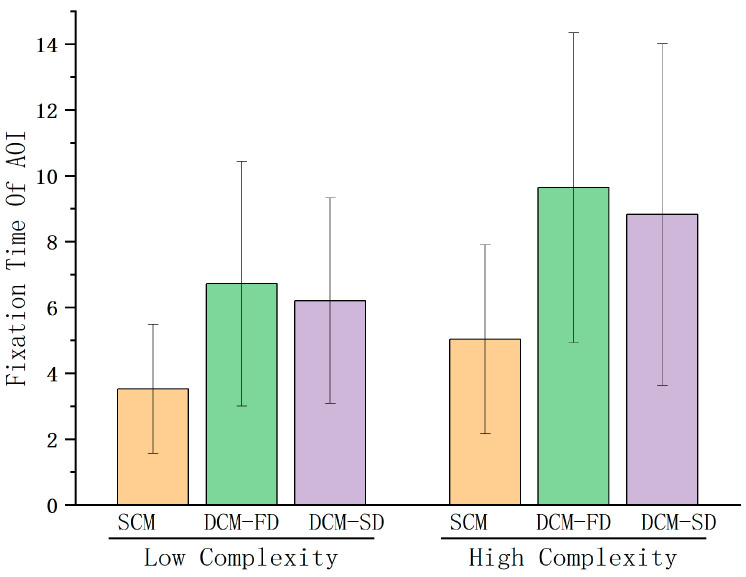
Fixation time depending on laying task complexity in three groups.

Considering the laying task complexity, the fixation time of the three groups was found to be significantly different (as shown in Table 5) based on the one-way ANOVA. Tukey’s HSD post hoc test comparisons were subsequently conducted and showed no significant pairwise differences between any groups (Figure 7). The fixation time of the SCM group (mean = 3.522, SD = 1.961) was lower than that in the DCM-FD group (mean = 6.720, SD = 3.715, *p* = 0.053) and the DCM-SD group (mean = 6.202, SD = 3.120, *p* = 0.098) in the low-complexity laying task. In the high-complexity laying task, the fixation time of the SCM group (mean = 5.034, SD = 2.869) was also lower than that in the DCM-FD group (mean = 9.639, SD = 4.719, *p* = 0.057) and the DCM-SD group (mean = 8.824, SD = 5.188, *p* = 0.114).

#### 4.2.3. Number of Fixations

As shown in Table 2, a statistically significant difference in the number of fixations was observed among the three groups based on one-way ANOVA (F(2, 30) = 8.011, $\eta_{p}^{2}$ = 0.348, *p* = 0.002). Post hoc analysis with Tukey’s HSD revealed that the number of fixations was significantly lower in the SCM group (mean = 69.182, SD = 26.168) compared with the DCM-SD group (mean difference = −41.151, SD = 10.282, Cohen’s d = −1.671, 95% CI [−2.630, −0.711], *p* = 0.001).

Considering the task complexity, the number of fixations of the three groups is shown in Table 6. No significant difference was observed among the three groups in both the low-complexity laying (F(2, 30) = 2.133, $\eta_{p}^{2}$ = 0.125, *p* = 0.136) and high-complexity laying tasks (F(2, 30) = 1.257, $\eta_{p}^{2}$ = 0.077, *p* = 0.299). However, the number of fixations showed the same trend for both low- and high-complexity laying tasks; i.e., SCM group < DCM-FD < DCM-SD.

### 4.3. Perceived Workload (NASA-TLX)

**Figure 8:**
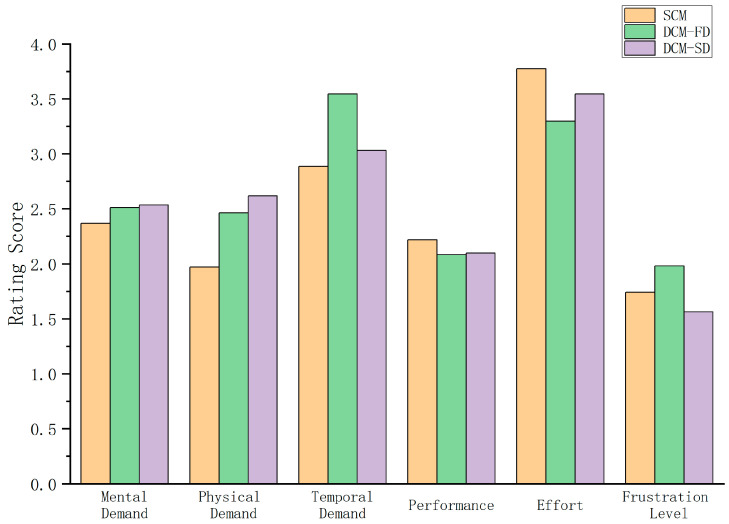
NASA Raw TLX score of each dimension in three groups.

After the experiments, the subjective workload perceived by the participants was measured across the six dimensions of the NASA-TLX, i.e., mental demand, physical demand, temporal demand, performance, effort, and frustration level. Each dimension was rated on a seven-point scale (1 = very low; 7 = very high), with higher scores indicating a higher workload in this study. Figure 8 shows the NASA Raw TLX score chart of the six dimensions for the three groups.

Participants across all three groups reported a low level of perceived workload in all dimensions (max. mean = 3.547 in effort), where a floor effect was observed and will be discussed in the later section. The data did not meet the assumptions of normality. Thus, the nonparametric Kruskal–Wallis test was performed to examine the differences among groups. The results show no significant difference in the perceived workload among the three groups (H(2) = 0.050, *p* = 0.975), with a small effect size (ε^2^ = 0.001, 95% CI [0.000, 0.081]) in this study. No significant differences in the other dimensions of NASA-TLX were observed among groups.

## 5. Discussion

Most ARAA research studies on wiring tasks are concerned with differences in performance and workload between AR instructions and paper-based manual methods. In these studies, the AR instructions achieved better task performance than paper-based instructions (Fang et al., 2025; Geng et al., 2024; Papetti et al., 2023; Rice et al., 2016; Szajna et al., 2020). Based on these previous findings, this study focused on the wiring operation to investigate how three kinds of AR visual modes support novice operators in completing tasks effectively. Due to the differences in characteristics between rigid-part mechanical assembly and the wire harness assembly processes, some distinctive results were found for the formboard wiring task when different AR visual modes were used in our controlled laboratory environment.

### 5.1. AR Mode Impact on Wiring Assembly Task Performance

#### 5.1.1. Task Time

Although wiring harness assembly on a formboard can be classified as a repetitive skill-based job that can be performed with basic training (J. Li et al., 2024), the laying of multiple wire harnesses requires the memorization of many bending points. The operators cannot make mistakes under the required work cycle, which puts high demands on the operator’s memory load. The AR-based real-time presentation of the wire path can effectively reduce the memory burden of operators and improve the work efficiency compared with paper-based manual methods (Papetti et al., 2023). Our study found a reversal relative to prior findings in rigid-part mechanical assembly tasks when different AR instruction modes were used during the wiring operation on a formboard.

Although the one-way ANOVA revealed a potentially significant main effect of the groups (*p* = 0.046) in total task time (Table 2), Tukey’s HSD post hoc test did not identify statistically significant pairwise differences (Figure 4a). This outcome may be attributed to the conservative nature of Tukey’s HSD test, which applies strict correction for multiple comparisons. In addition, the relatively small sample size in this study may have reduced the statistical power for detecting pairwise differences, even though the overall group difference was significant. However, overall, the average task time of the SCM group was on average 18% faster than that of the other two groups. The obtained results are different from past research work on rigid-part mechanical assembly tasks, where the AR animation modes achieved a higher task performance (Hou et al., 2013, 2015; Lušić et al., 2016; Smith et al., 2020; Laviola et al., 2025). For rigid-part mechanical assembly, the parts occupy specific 3D positions within the final products. The parts assembly process should be clearly understood in 3D space (Laviola et al., 2025). The AR animation modes were found to be more understandable for learning the assembly process even if they required more cognitive workload (Deshpande & Kim, 2018; Drouot et al., 2022; Z. Yang et al., 2019). Meanwhile, for wiring tasks on a formboard, the wiring routing paths and clamps they pass through are more important (Caudell & Mizell, 1992; Papetti et al., 2022, 2023). The spatial relationships are not complex to understand; the key information required for wire harness assembly mainly includes the wire color and wire routing direction. The static mode of the SCM group experienced no changes and could directly display the color and routing direction. The blinking model of the DCM-FD group needed to blink at least once for its changes to be perceived. The segment-by-segment mode of the DCM-SD group required all steps to be displayed before operators could understand the overall routing direction. Compared with the flash and segment wire path modes, the static wire path modes in the SCM group had the advantage of seeing everything at a glance, which enabled faster cognition during wiring tasks.

We analyzed low- and high-complexity laying tasks separately, as shown in Table 3 and Figure 4b. No significant difference was detected among the three groups when considering the task complexity alone. We did not change the flashing speed and segment display speed during the experiments. It is reasonable to assume that, if the two speeds were to be reduced, operators would need more time while waiting to obtain the required path information. Compared with the flash and segment wire path modes, the assembly task time of the SCM group with static wire path modes was approximately 11% lower than that of the other two groups in both low- and high-complexity tasks. In summary, regardless of whether it was the total task time or the time taken to complete tasks of a different complexity, the SCM groups had advantages over the dynamic groups (DCM-FD and DCM-SD) under the single-route conditions used in this study. A lower assembly task time is an important factor for customized wire harness production with a small batch size (Trommnau et al., 2019). In addition, based on our experimental results, the SCM instructions are of potential significance for improving assembly efficiency even if the participants were all novice operators, when considering the single-route conditions applied in this study experiments.

Although this study was limited by the small sample size and no statistically significant pairwise differences were detected via Tukey’s HSD post hoc test for total assembly task time, the large effect sizes of the ANOVA ($\eta_{p}^{2}$ = 0.186) indicated that there were significant overall differences among the three groups (*p* = 0.046). Therefore, H1 (the visual style of the wire harness mode has a significant effect on the task time during the ARAA process for novice operators) cannot be fully accepted in our controlled laboratory experiments regarding the total assembly task time. The data provide evidence for an overall group effect, but fail to confirm significant differences between specific visualization modes. When analyzing low- and high-complexity laying tasks separately, the visual style of the wire harness mode showed no significant effect on assembly time under the current experimental setup.

#### 5.1.2. Number of Assembly Errors

Regarding the number of assembly errors, the three groups exhibited a relatively low total number of errors (mean = 0.182 in SCM, mean = 0.300 in DCM-FD, mean = 0.083 in DCM-SD) as the simple wiring task required only a short assembly task time. The results are consistent with the literature (Papetti et al., 2023; Fang et al., 2024; Brunzini et al., 2024) that ARAA methods could reduce assembly errors. Notably, the DCM-SD group had the lowest number of wire assembly errors despite the longer assembly task time. The reason may be that the segment-by-segment display of the AR wire path mode can intuitively and clearly provide the current bending points of each segment to the participants, which enables them to easily distinguish the path of the wires and make less errors. Such influences have yet to be explored in both wire-harness ARAA (Papetti et al., 2022, 2023; Fang et al., 2024; Brunzini et al., 2024) and rigid-part ARAA (Smith et al., 2020; Ariansyah et al., 2022; Arige et al., 2022; Laviola et al., 2025).

The overall number of assembly errors was low, indicating that the task was relatively easy or that the participants possessed high proficiency in this simple task. This resulted in a lack of variance in the number of assembly errors, which may have limited the observation of the potential effects of the AR mode visual style. In the current experimental study, there were no significant differences in the number of assembly errors among the three groups (*p* = 0.434) with a small effect size (ε^2^ = 0.018), and the 95% confidence interval [0.000, 0.068] included zero. This might indicate that the actual differences among the three groups were minimal, and the grouping factor had almost no substantial effect on the observed indicators. H2 (the visual style of the wire harness mode has a significant effect on the number of assembly errors during the ARAA process for novice operators) is therefore not supported by the current controlled laboratory study.

### 5.2. AR Mode Impact on Assembly Gaze Behavior and Users’ Experience

#### 5.2.1. Gaze Behavior

Eye movement behavior can reflect the cognitive process of an operator, objectively reflecting the operator’s attention to the AOIs and their information needs during complex manufacturing tasks (Suzuki et al., 2024). The total fixation time reflects the duration of visual attention allocation. The number of switches characterizes the pattern of visual exploration. The number of fixations reflects the frequency of visual search and the efficiency of information extraction. For the formboard wiring task investigated in this study, the key cognitive task was to understand the routing path and corresponding clamps for each wire. The analysis of eye movement during the wire-laying tasks might suggest that the participants devoted more effort to interpreting dynamic instructions (DCM-FD and DCM-SD) relative to static instructions (SCM) across all three eye movement indicators.

As shown in Table 2, there were no statistically significant differences (*p* = 0.058, $\eta_{p}^{2}$ = 0.173) in the number of switches across different wire visualization modes for the three groups during the assembly process of the whole eight wires. Considering the number of switches between different AOIs, our finding is consistent with a previous ARAA study on rigid-part mechanical assembly (X. Yang et al., 2023) in which longer switching times were needed in the AR animation groups. However, in other ARAA research studies on rigid-part mechanical assembly using eye movement data (Lušić et al., 2016; Laviola et al., 2025), the dynamic AR information delivery reduced the number of additional gazes at instructions, which means that animated modes can provide a clearer task description regarding the correct component orientation. These varying, even conflicting, results stem from the use of different assembly objects/tasks, operator personalities, and mediums that present augmented information (Buchner et al., 2022; B. Wang et al., 2024; Fang et al., 2025), e.g., the HMD, glasses, projector, and display screen. For the 2D path-finding laying task, the static path display potentially reduces the interference caused by dynamic visualization and improves memory efficiency, which results in less switching times between different AOIs.

During the laying phase, the static mode of the SCM group directly presents the overall routing information at one time and more required information can be obtained in a single view. The blinking effect of the DCM-FD group is mainly achieved through variations in the thickness of the wire-path. In the segment-by-segment mode of the DCM-SD group, operators can only obtain the required local path when the specific segment is displayed. The characteristics of the three AR modes led to the number of switches in the dynamic-mode groups (DCM-FD) being significantly higher than that in the SCM group (*p* = 0.031) for the laying tasks. The number of switches in the SCM group was also significantly lower than that of the DCM-FD group in the low- (*p* = 0.041) and high-complexity laying tasks (*p* = 0.037), as shown in Figure 5.

Figure 6 shows that fixation times for the total task and the two sub-tasks on the AR display differed significantly among the three groups. In the SCM group, the static mode exhibited no visual disturbance due to its unchanging state, enabling operators to reduce the fixation time on the display by 7.621 and 6.289 s compared with the DCM-FD and DCM-SD groups, respectively. In the DCM-FD group, the blinking mode necessitates a minimum of one blinking cycle to transmit wire information, thereby requiring a relatively extended gaze duration. For the segment-by-segment mode in the DCM-SD group, operators needed to wait until the model had rendered the specific local position required to obtain the routing information.

In Table 5, the total fixation time of the three groups might suggest a significant difference regardless of whether the tasks had a low (*p* = 0.041) or high complexity (*p* = 0.047). Although Figure 7 shows that no statistically significant pairwise differences were detected following Tukey’s HSD post hoc test, the large effect sizes of the ANOVA ($\eta_{p}^{2}$ = 0.191 for the low-complexity tasks, $\eta_{p}^{2}$ = 0.184 for the high-complexity tasks) for fixation times indicated that there were significant overall differences among the three groups considering the laying task complexity. With an increasing task complexity, a longer fixation time on the AR information was observed, similarly to the study by Bläsing and Bornewasser (2021). For all assembly scenarios in this study, the SCM group had the lowest fixation time for wiring tasks. For the number of fixations shown in Table 2, the SCM group exhibited a significantly lower number of fixations compared with the DCM groups, potentially indicating that the SCM visual style reduced visual searching and achieved more efficient information extraction in the wiring task on a formboard. These results are different from those of the study (Laviola et al., 2025), where static AR information required longer fixation times and a higher number of fixations during the orientation task of rigid-part mechanical assembly, which means more cognitive load was needed (Kosch et al., 2023). The findings of this study are consistent with those reported in previous research (Ariansyah et al., 2022; Biondi et al., 2023; Stork & Schubö, 2010), where eye movement analysis showed that a longer fixation time, together with a greater number of switches and more fixations, resulted in longer task times.

Based on the above results, H3 (the visual style of the wire harness mode has a significant effect on the gaze behavior during the ARAA process for novice operators) was provisionally confirmed by our controlled laboratory experiments, considering the number of switches in the laying phase (*p* = 0.041, $\eta_{p}^{2}$ = 0.192), the total fixation time on the AR display (*p* = 0.003, $\eta_{p}^{2}$ = 0.322), and the number of fixations on AOIs (*p* = 0.002, $\eta_{p}^{2}$ = 0.348).

#### 5.2.2. Perceived Workload (NASA-TLX)

Considering the subjective workload results, all three groups had low overall NASA-TLX scores, showing a floor effect. On the one hand, the low scores indicated a reduced perceived workload among operators in the three ARAA groups, confirming the findings reported in the literature (Hou et al., 2013, 2015; Zhao et al., 2021; Marino et al., 2024; Fang et al., 2024; T. Zhang et al., 2025; Lucchese et al., 2025). On the other hand, the clustering of NASA-TLX scores for each dimension near the floor (max mean = 3.547 in SCM group for effort, min mean = 1.563 in DCM-SD for frustration level) limited the sensitivity to detect workload differences, and, thus, it was difficult to detect a significant difference across the three AR instruction modes.

From the perspective of the mental demand incurred by decision-making, memory retrieval, information processing, and precise judgment, an increased volume of information corresponds to a higher level of mental workload for an operator (Hou et al., 2013). The information presented by the three AR modes did not require much mental demand (95% CI [2.061, 2.883]) and therefore did not cause the participants to experience information overload. As a simple repetitive skill-based job with basic training (J. Li et al., 2024), the physical demand (95% CI [1.899, 2.812]) of the wiring task on a formboard was low for the eight-wire assembly experiment. In the simple assembly task with AR visual instruction, the participants reported a good task performance (95% CI [1.666, 2.600]), less effort (95% CI [2.953, 4.141]), and lower frustration (95% CI [1.412, 2.087]).

Based on the current experimental results, H4 (the visual style of the wire harness mode has a significant effect on users’ subjective experience for novice operators) is not supported by our controlled laboratory experiments (*p* = 0.975, ε^2^ = 0.001). This might suggest that all experimental conditions provided a relatively friendly and low-workload experience. The narrow confidence intervals further reflected strong inter-participant consistency and robust, reliable subjective responses. Longer-task-duration and more complex wiring tasks should be conducted in the future to test for subtle differences during novice training.

### 5.3. Analysis of Retained and Excluded Participants

The number of excluded participants (low sampling rates of eye-tracking data) varied substantially across the three experimental groups (4, 5, and 3 exclusions, respectively, from the initial 15 subjects per group), resulting in unequal final sample sizes (11, 10, and 12) and uneven attrition rates. To verify whether data bias was introduced through participant exclusion procedures, core observational indicators were compared between the retained valid participants and excluded abnormal participants. Due to the small sample sizes in the retained and excluded groups, the exact Mann–Whitney U test was adopted instead of the normal approximation. Between-group differences were quantified using Cliff’s δ and bootstrapped 95% CIs.

Table 7 shows the descriptive statistics of the retained and excluded groups across the three AR modes for the total task time, number of assembly errors, and NASA-TLX scores. The results reveal no statistically significant distributional differences in the examined metrics between the retained and excluded groups based on the observed data (all *p* > 0.05), where effect sizes varied in magnitude for the different indicators of the three AR modes. For the total task time, only the between-group difference in the DCM-SD mode shows a moderate effect size (|Cliff’s δ| = 0.389 > 0.330). For the NASA-TLX scores, only the between-group difference in SCM mode shows a moderate effect size (|Cliff’s δ| = 0.409 > 0.330). These results imply a practically meaningful difference between the retained and excluded group from a substantive perspective. Nevertheless, the statistical test returned a *p*-value above 0.05, and the 95% CI fully spanned zero, failing to support a statistically reliable group distinction. Due to the small sample sizes of the retained and excluded groups, we cannot rule out the potential group divergence solely based on the obtained non-significant results. For the number of assembly errors, Cliff’s δ effects for the retained and excluded group across the three AR modes fall within a negligible magnitude (|Cliff’s δ| < 0.147). The 95% CIs fully cross zero or lie close to zero. This result indicates that the risk of sample bias introduced by excluding participants with assembly errors is extremely low.

### 5.4. AR Device Configurations in Visual Wiring Operation

In this study, a monitor-based AR configuration was selected as the visualization method of the wire models. It is well-known that there are three types of device configurations for ARAA (Danielsson et al., 2020; Eswaran et al., 2023), i.e., wearable devices (HMDs and smart glasses), hand-held devices (tablets and smart phone) and fixed-mounted displays (monitor-based and projection-based). Wearable AR devices have emerged as a prominent solution for ARAA due to the inherent mobility, hands-free operation, and abundant built-in natural interaction capabilities. However, they remain in the early stage of practical deployment (Fang et al., 2025; B. Wang et al., 2024; Danielsson et al., 2020) because of their restricted field of view, limited battery life, weight load, and visual impairment induced over continuous use (Drouot et al., 2021).

For a fixed location with a limited work area, such as a wire-harness formboard station, fixed-mounted monitor displays are a suitable AR configuration because of their head-hands-free operation, lack of a physical workload, and simple setup, requiring minimal workstation renovation (Alves et al., 2022; Angel et al., 2022; Cardoso et al., 2020; Z. Yang et al., 2019). The review by B. Wang et al. (2024) showed that 14% of studies used display screens for the visualization configuration. For the wiring operation on the formboard, Papetti et al. (2023) conducted laboratory comparative experiments based on a monitor, HMD, and projection display. Instructions presented on the monitor display were rated the most understandable, as reflected in the lower error rate. Operators were more satisfied with their task performance regarding both time efficiency and ease of task execution when following the monitor display. Therefore, the AR experimental configuration in this study is applicable to practical wire harness assembly workstations. Apparently, fixed-mounted AR displays are constrained by the mounting height, effective work area, and camera focal length (Fang et al., 2025). The decision to use these in industrial environments should be based on an evaluation of the spatial constraints, the length of the wire harness assembly, and dynamic operation requirements, which are not the focus in this study.

Our work is an exploratory laboratory study that examined the impacts of AR instruction modes on assembly performance for specific wiring operations, and not a general AR design evaluation for common industrial application. Different ARAA devices exhibit distinct characteristics and potential advantages in specific use cases (Alves et al., 2022; Haas et al., 2024). Prior studies have shown that distinct AR device configurations produce differences in user behavior and performance, even when adopting the same visual modes (Alves et al., 2022; Haas et al., 2024). In our study, the monitor-based AR display resulted in a switch cost, which is also a common load concern in the HMD-based independent virtual screen AR display method (X. Yang et al., 2023). With an HMD-based in situ real–virtual fusion AR display, although no switch cost is induced, the permanently displayed visual information in the field of view may cause visual distraction, induce a higher cognitive load, and, thus, reduce operational performance (Szajna et al., 2020; Danielsson et al., 2020; Biondi et al., 2023). Regarding the generalization and comparison of our research results to HMD AR setups, future experimental work should be conducted.

### 5.5. Limitation and Future Work

This study has several limitations concerning participants, experimental conditions, and the parameters of the wire AR modes.

Firstly, all participants were college students who were novice operators in wiring activities, and the experiments were conducted in a controlled lab environment. No experienced operators were involved. Age, prior experience, technological familiarity, individual abilities (e.g., manual dexterity, and planning competence), and training strategies are key factors significantly influencing task performance and error rates (Lucchese et al., 2026). Accordingly, the results observed in this study are mainly applicable to novice operators. Experienced expert operators may rely less on external guidance and more on internalized templates, potentially reducing the relative advantage of dynamic AR visualization (Funk et al., 2017). It is necessary to test the effects of AR wiring modes in a shop floor environment with operators who have different skill levels and experiences.

Secondly, each participant’s experimental tasks only lasted for a short time period on a simple task. The duration of our experimental tasks failed to account for the long-term fatigue and learning effects characteristic of 8 h work shifts. Additionally, the controlled lab environment conditions may not be adequate for fully replicate real-world industrial settings, which are often characterized by noise, lighting, and time-related pressure. From a physical ergonomics perspective, the fatigue in the lower limbs caused by standing for longer periods of time and the influence of the tilt angle of a wiring formboard (Papetti et al., 2023) on the operators in industrial sites were not considered in this study. Experimental scenarios closer to industrial reality can improve measurement sensitivity. Testing operators across different skill levels under these more demanding conditions may help us detect subtle differences that were not captured in this study. Such designs would reduce floor or ceiling effects and allow for a more rigorous evaluation of performance differences. For these reasons, the findings from experiments conducted in the controlled lab environment of this study cannot directly transfer to skilled industrial operators in the shop floor.

Thirdly, only a fixed flashing rate in the DCM-FD group and a fixed segment progression speed in the DCM-SD group were used in our experiments. These two parameters of the dynamic wire modes are important for the timing differences during the assembly processes. In our study, the same animation settings were adopted for all groups, yielding consistent effects and trends in the results. Notably, previous research on dynamic ARAA (Hou et al., 2013, 2015; Lušić et al., 2016; Deshpande & Kim, 2018; Moghaddam et al., 2021; Ariansyah et al., 2022) did not present the animation rate or speed parameters. Laviola et al. (2025) mentioned that the cycle time for their part assembly path animation time was 3 s. Given that these parameters may directly influence the waiting time and gaze allocation, the findings of this study should be interpreted as valid only under the specific parameters tested in this study. To further explore the sensitivity of the animation parameters to the task performance, additional experiments tailored to this specific wiring application scenario are required, which will be an interesting and indispensable research pathway in the ARAA industrial application field.

Our current work represents only the beginning of ARAA evaluation in industrial wire harness assembly on a formboard. In our experiments, all participants completed tasks in a fixed order, which may introduce subtle practice effects on their behavior and performance. Although pre-training and task heterogeneity minimized such bias, a fully randomized task sequence is recommended for subsequent studies to eliminate sequence-related confounding factors. In the future, assessing long-term performance will also provide key insights into the AR effectiveness and its acceptance in practical use scenarios. In the wiring of complex wire products, the laying task should consider the start and end clamps of wires that cannot be reversed. Specifically, how to use the AR mode or other virtual assets to match the start clamps is left for future work. In this study, the wiring task was relatively simple. In the experiment, all wire harness paths were designed as single routes without branches. The inherent advantage originally designed for DCM-SD, namely, step-by-step segment guidance, is difficult to achieve under such conditions. Exploring the effects of three types of AR modes on a wire bundle with branches is also interesting and valuable work. The main manual wiring activities performed on a formboard include clamp placement, wire laying, quality checks (whether the correct wires are clustered for a single connector based on the wires’ serial number), and bundle protection. In future work, the experiments will be extended to cover different physical wiring activities, aiming to observe which activity benefits more from different AR wire path modes. With the popular use of Electroencephalography (EEG) and Near-Infrared Brain Functional Imaging (fNIRS) in cognitive load recognition (Atici-Ulusu et al., 2021), more objective indicators of workload can be obtained to analyze the AR modes’ effect on the wiring assembly process. This is also a direction for future work.

## 6. Conclusions

In the Industry 5.0 era, AR instructions will support the operator assembly processes, thereby enhancing efficiency and reducing the cognitive load in smart factories. The purpose of this exploratory laboratory study was to investigate task performance, gaze behavior, and subjective experience with three wire path AR modes during the assembly process on a formboard. Eye-tracking and a post-experiment questionnaire were employed to evaluate novice operators training in a controlled laboratory environment. A between-subjects experiment was conducted based on the monitor display of the AR wire path models under single-route conditions. The findings might suggest that an SCM can help operators achieve the fastest task time, and significant overall differences were observed among the three groups under the specific settings used in this study. The number of switches in the SCM group tended to be lower than that in the DCM-FD groups for both low- and high-complexity wiring tasks during the wire-laying phase. The total fixation time on the AR display and the number of fixations among the three groups showed notable differences, with the SCM group exhibiting the lowest values. These results indicate that animation modes (DCM-FD and DCM-SD) can increase the fixation behavior and result in a longer task time. Meanwhile, a significant effect on the number of assembly errors was not observed for the visual style of the wire harness mode in the controlled lab environment. In addition, subjective experiences did not show significant differences among the three groups in this simplified, short, and repetitive wiring task. Our findings provide insight into the visual design of ARAA wiring systems for novice operators and can serve as references for future studies on the industrial scenarios with complex manual wiring tasks.

## Figures and Tables

**Table 1:** The wire assembly tasks with different color modes, segments, and clamps.

Wires No.	1	2	3	4	5	6	7	8
Color	Purple	Orange	Purple	Purple	Orange	Purple	Orange	Orange
Segments	4	2	3	3	2	4	2	2
Number of clamps	7	4	7	7	4	7	4	4
Complexity	High	Low	High	High	Low	High	Low	Low

**Table 2:** Descriptive statistics for dependent variables of three groups with different visual modes.

Variables	SCM		DCM-FD		DCM-SD		F	N	*p*	$\eta_{p}^{2}$ [95% CI]
	Mean	SD	Mean	SD	Mean	SD				
Total task time (s)	99.555	18.970	116.987	15.734	116.812	18.611	3.431	33	0.046	0.186 [0.002, 0.370]
Number of assembly errors	0.182	0.405	0.300	0.483	0.083	0.289	-	33	-	-
Number of switches	66.909	18.070	83.800	11.094	76.583	16.138	3.139	33	0.058	0.173 [0.000, 0.352]
Fixation time (s)	13.436	5.842	24.217	8.901	26.386	10.488	7.123	33	0.003	0.322 [0.086, 0.516]
Number of fixations	69.182	26.168	90.100	17.953	110.333	27.720	8.011	33	0.002	0.348 [0.112, 0.502]
NASA-TLX	2.493	0.883	2.647	1.166	2.565	1.043	-	33	-	-

**Table 3:** Statistics of assembly time in three groups with laying task complexity.

Task Complexity	SCM		DCM-FD		DCM-SD		F(2, 30)	*p*	$\eta_{p}^{2}$ [95% CI]
	Mean	SD	Mean	SD	Mean	SD			
Low	31.335	7.838	35.805	5.111	35.489	7.334	1.416	0.259	0.086 [0.000, 0.267]
High	42.402	9.413	49.044	6.974	47.869	10.744	1.571	0.225	0.095 [0.000, 0.279]

**Table 4:** Statistics of number of switches in three groups with laying task complexity.

Task Complexity	SCM		DCM-FD		DCM-SD		F(2, 30)	*p*	$\eta_{p}^{2}$ [95% CI]
	Mean	SD	Mean	SD	Mean	SD			
Low	22.182	7.718	29.300	3.917	26.500	6.613	3.359	0.048	0.183 [0.002, 0.368]
High	35.273	10.403	45.700	8.028	38.412	8.867	3.531	0.042	0.191 [0.003, 0.376]

**Table 5:** Statistics of fixation time in three groups with laying task complexity.

Task Complexity	SCM		DCM-FD		DCM-SD		F(2, 30)	*p*	$\eta_{p}^{2}$ [95% CI]
	Mean	SD	Mean	SD	Mean	SD			
Low	3.522	1.961	6.720	3.715	6.202	3.120	3.548	0.041	0.191 [0.006, 0.350]
High	5.034	2.869	9.639	4.710	8.824	5.188	3.389	0.047	0.184 [0.007, 0.338]

**Table 6:** Statistics of number of fixations in three groups with laying task complexity.

Task Complexity	SCM		DCM-FD		DCM-SD		F(2,30)	*p*	$\eta_{p}^{2}$ [95% CI]
	Mean	SD	Mean	SD	Mean	SD			
Low	18.818	11.557	22.900	6.350	26.750	8.740	2.133	0.136	0.125 [0.000, 0.316]
High	28.817	14.400	36.900	10.999	37.083	15.664	1.257	0.299	0.077 [0.000, 0.255]

**Table 7:** Descriptive statistics of retained and excluded participants in three AR modes.

Variables	AR Modes	Retained			Excluded			*p*	Cliff’s δ [95% CI]
		Mean	SD	*n*	Mean	SD	*n*		
Total task time	SCM	99.555	18.970	11	104.778	7.239	4	0.661	−0.182 [−0.727, 0.409]
	DCM-FD	116.987	15.734	10	110.889	12.971	5	0.635	−0.020 [−0.536, 0.812]
	DCM-SD	116.812	18.611	12	104.288	7.551	3	0.312	0.389 [−0.167, 0.833]
Number of assembly errors	SCM	0.182	0.405	11	0.500	1.000	4	0.640	−0.114 [−0.705, 0.364]
	DCM-FD	0.300	0.483	10	0.500	1.000	5	0.929	−0.025 [−0.675, 0.500]
	DCM-SD	0.083	0.289	12	0.000	0.000	3	0.617	0.083 [0.000, 0.250]
NASA-TLX	SCM	2.493	0.883	11	2.135	1.467	4	0.239	0.409 [−0.545, 0.983]
	DCM-FD	2.647	1.166	10	2.875	0.370	5	0.357	−0.325 [−0.825, 0.250]
	DCM-SD	2.566	1.041	12	2.444	0.918	3	0.828	0.094 [−0.611, 0.778]

## Data Availability

The data are not publicly available due to privacy restrictions, as they include responses from human participants.

## Appendix A

**Table A1:** Wiring layouts of eight wires in the experiments *.

1	2	3	4

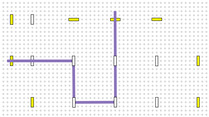
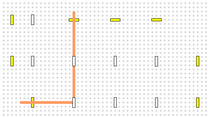
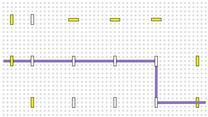
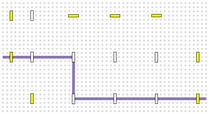
			
5	6	7	8

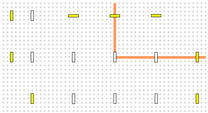
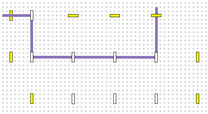
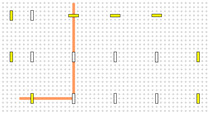
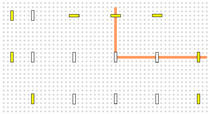
			

* The small rectangles indicate the positions and orientations of wire clamps. Each wire has a unique routing path with distinct starting and terminal clamps.
